# Response of fibroblast growth factor 19 and bile acid synthesis after a body weight-adjusted oral fat tolerance test in overweight and obese NAFLD patients: a non-randomized controlled pilot trial

**DOI:** 10.1186/s12876-018-0805-z

**Published:** 2018-06-04

**Authors:** Dana Friedrich, Hanns-Ulrich Marschall, Frank Lammert

**Affiliations:** 1 Department of Medicine II, Saarland University Medical Center, Saarland University, 66421 Homburg, Germany; 2 Department of Molecular and Clinical Medicine, Sahlgrenska Academy, Institute of Medicine, University of Gothenburg, Gothenburg, Sweden

**Keywords:** Bile acids, FGF19, Non-alcoholic fatty liver disease, Oral fat tolerance test

## Abstract

**Background:**

Non-alcoholic fatty liver disease (NAFLD) is common both in obese and overweight patients. Fibroblast growth factor 19 (FGF19), an intestinal hormone, could play a role in the complex pathogenesis of NAFLD. The aim of our study was to investigate responses of FGF19 and bile acid (BA) synthesis after a body weight-adjusted oral fat tolerance test (OFTT) in overweight and obese NAFLD patients.

**Methods:**

For this study, we recruited 26 NAFLD patients; 14 overweight (median BMI 28.3 kg/m^2^), 12 obese (35.3 kg/m^2^) and 16 healthy controls (24.2 kg/m^2^). All individuals received 1 g fat (Calogen®) per kg body weight orally. Serum concentrations of FGF19 were determined by ELISA. Concentrations of BAs and BA synthesis marker 7α-hydroxy-4-cholesten-3-one (C4) were measured by gas chromatography-mass spectrometry and high-performance liquid chromatography, respectively; all at 0 (baseline), 2, 4 and 6 h during the OFTT.

**Results:**

BMI correlated negatively with fasting FGF19 concentrations (rho = − 0.439, *p* = 0.004). FGF19 levels of obese NAFLD patients were significantly (*p* = 0.01) lower in the fasting state (median 116.0 vs. 178.5 pg/ml), whereas overweight NAFLD patients had significantly (*p* = 0.004) lower FGF19 concentrations 2 h after the fat load (median 163.0 vs. 244.5 pg/ml), and lowest values at all postprandial time points as compared to controls. Baseline BA concentrations correlated positively with FGF19 values (rho = 0.306, *p* = 0.048). In all groups, we observed BA increases during the OFTT with a peak at 2 h but no change in C4 levels in overweight/obese NAFLD patients.

**Conclusions:**

Reduced basal gastrointestinal FGF19 secretion and decreased postprandial response to oral fat together with blunted effect on BA synthesis indicate alterations in intestinal or hepatic FXR signaling in overweight and obese NAFLD subjects. The precise mechanism of FGF19 signaling after oral fat load needs further evaluation.

**Trial registration:**

We have registered the trial retrospectively on 30 Jan 2018 at the German clinical trials register (http://www.drks.de/), and the following number has been assigned DRKS00013942.

**Electronic supplementary material:**

The online version of this article (10.1186/s12876-018-0805-z) contains supplementary material, which is available to authorized users.

## Background

Obesity and fatty liver disease represent increasing medical problems in developed countries. In Germany, the prevalence of obesity increased during the years 1998 to 2011 from 18.9 to 23.3% in men and from 22.5 to 23.9% in women [1]. In the United States, 37% of adults are obese [2]. Obesity is an important risk factor of non-alcoholic fatty liver disease (NAFLD), which has been reported in 30 to 40% of adults [3, 4].

The term NAFLD is used for a wide spectrum of fatty liver diseases that starts with simple steatosis in non-alcoholic fatty liver (NAFL) that may progress to non-alcoholic steatohepatits (NASH), which is complicated by fibrosis, cirrhosis, and eventually hepatocellular carcinoma [5–7]. NAFLD is often associated with the metabolic syndrome and requires exclusion of excessive alcohol consumption as well as viral and autoimmune liver diseases [8]. NAFLD is common in obesity but also in overweight patients [9, 10]. The pathophysiology of NAFLD is complex and still not fully defined [11, 12]. Several metabolic factors have already been identified in the development of NAFLD, including insulin resistance, diabetes mellitus and obesity.

So far there have been only few studies of the importance of gastrointestinal hormones in the pathogenesis of NAFLD [13–15]. The gastrointestinal hormone fibroblast growth factor 19 (FGF19) has emerged as a novel regulator of bile acid, carbohydrate and lipid metabolism. In human metabolic syndrome associated diseases, such as type 2 diabetes mellitus (T2DM) and NAFLD, FGF19 signaling seems to be dysregulated [16]. In animals, FGF19 transgenic mice show resistance to a high-fat diet and decreased liver triglyceride concentrations [17] while the administration of recombinant FGF19 increases the metabolic rate [18]. Accordingly, in humans with NAFLD, reduced fasting FGF19 levels were found [14, 19, 20]. Therefore, the present study focuses on the dietary regulation of FGF19 and its potential role in the pathogenesis of NAFLD.

FGF19 release in the intestine is induced by bile acids (BAs). After a meal, the entry of dietary fat in the duodenum causes gallbladder contraction and BA inflow into the intestinal lumen. The reabsorption of BAs in the terminal ileum activates the canonical BA sensor farnesoid X receptor (FXR), resulting in enhanced transcription and secretion of FGF19 [21, 22]. FGF19 binds on hepatocytes to the FGF receptor 4 (FGFR4) and its cofactor βKlotho [22–24], which triggers a signaling cascade that represses cholesterol 7α-hydroxylase (CYP7A1), the rate-limiting enzyme in BA synthesis from cholesterol [25]. 7α-Hydroxy-4-cholesten-3-one (C4) is an intermediate of BA synthesis, which can be measured in serum [26].

Since the role of FGF19 in the pathogenesis of human NAFLD is unknown, we studied FGF19 and hepatic downstream effects (C4 and BAs) in overweight and obese NAFLD outpatients (and healthy controls) that were subjected to a body weight-adjusted oral fat tolerance test (OFTT). We determined serum concentrations of FGF19, C4 and BAs at baseline and at 2, 4 and 6 h after OFTT. We hypothized that FGF19 levels are lower in obese compared to overweight NAFLD patients.

We aimed to answer the following questions in this study:

1. Do fasting FGF19 serum concentrations differ between normal-weight healthy, overweight and obese NAFLD patients?
2. How does a body weight-adjusted oral fat tolerance test (OFTT) affect serum FGF19 concentrations in these populations?
3. How does a postprandial FGF19 response affect hepatic BA biosynthesis, as assessed by C4?

## Methods

### Study protocol

The study protocol was approved by the Ethics Committee of the Ärztekammer des Saarlandes, Saarbrücken (ID number 58/09). All subjects (≥ 18 years) were fully informed about the study objectives and methods and gave their written informed consent before participating in this non-randomized controlled pilot trial.

### Study subjects

During 2009 and 2010, we recruited overweight and obese NAFLD outpatients in the Department of Internal Medicine II, Saarland University Medical Center, Homburg, as well as healthy controls with normal body weight. Inclusion criteria for NAFLD were ultrasound and/or biopsy findings consistent with fatty liver disease. Exclusion criteria were increased alcohol consumption in medical history and the following acute and chronic liver diseases: cirrhosis, hepatitis A virus (HAV), hepatitis B virus (HBV), hepatitis C virus (HCV), hepatitis D virus (HDV), cytomegalovirus (CMV) and Epstein-Barr Virus (EBV) infections, hemochromatosis, Wilson’s disease, α_1_-antitrypsin deficiency, and autoimmune hepatitis. Healthy controls included employees of the clinic and medical students with normal BMI and no diseases in history. In controls, no liver and laboratory diagnosis was performed.

Subjects were divided into three groups according to their BMI (healthy controls, normal weight: 19.0–25.4 kg/m^2^, overweight NAFLD: 25.5–29.9 kg/m^2^, obese NAFLD: ≥ 30.0 kg/m^2^) [27, 28].

### Liver parameters

In overweight and obese patients (*N* = 26), liver status was assessed by abdominal ultrasound and/or liver biopsy. Ultrasound was performed using the Hitachi EUB-8500 ultrasound scanner (Hitachi Medical Systems, Wiesbaden, Germany). Hepatic steatosis results in abnormal echo patterns on ultrasound scanning; the severity of steatosis was graded as mild (I), moderate (II), or severe (III) [29]. Liver biopsy samples of five patients were examined by an experienced pathologist of Saarland University Medical Center.

### Oral fat tolerance test (OFTT)

We used a body weight-adjusted OFTT to investigate postprandial FGF19, BA and C4 responses in our study subjects. For the present study, a standardized test drink Calogen® (Nutricia, Erlangen, Germany) was administered, which is a lipid emulsion based on vegetable fat with 50% long-chain triglycerides [30]. All individuals received 1 g fat per kg body weight orally. The fat load was based on subjects’ body weight to adjust the OFTT to hypercaloric (especially high-fat) eating behavior of obese patients [31].

### Blood samples

Blood samples were drawn from a peripheral vein at 8:00 AM after an overnight fasting and 2, 4 and 6 h after the oral fat challenge. Samples were centrifuged for 10 min at 3000 g 30 min after blood collection (ROTANTA 46R, Hettich, Tuttlingen, Germany). Subsequently, serum was stored in aliquots at-70 °Cuntil analysis.

### Serum FGF19, bile acid and C4 measurements

FGF19 serum concentrations were measured in duplicate by quantitative sandwich enzyme-linked immunosorbent assay, using the FGF19 Quantikine ELISA kit (R&D Systems, Minneapolis, USA). Serum BA concentrations were determined by gas chromatography-mass spectrometry (GCMS) [32]. 7α-hydroxy-4-cholesten-3-one (C4), a valid marker of bile acid biosynthesis [33], was measured by high-performance liquid chromatography (HPLC).

### Statistical analysis

Data analysis was performed using SPSS (version 20.0, IBM, Ehningen, Germany). Kruskal-Wallis test was used to analyze quantitative data for differences within the cohort. For the present study with a low number of study subjects (*N* < 20), normal distributions were not expected [34]. Thus, data are expressed as medians and interquartile ranges (IQR 25–75). In addition, Mann-Whitney-U test was used to test differences between two groups. The strength of associations between two parameters was estimated using the non-parametric Spearman correlation test. Spearman’s correlation coefficient is presented as rho. For the OFTT, FGF19_(0-6h)_-area under the curve (AUC) and, after correcting for baseline, the incremental AUC (FGF19-IAUC) were computed using GraphPad Prism (version 6.0, GraphPad Software, La Jolla, CA, USA). A *p*-value < 0.05 denotes statistical significance.

## Results

### Subject characteristics

Table 1 summarizes the subject characteristics. A total of 42 subjects, 21 women and 21 men, were recruited for our study. Study participants were between 19 and 68 years old (median 47.0 years, IQR 28.8–53.8). Overall, we recruited 14 overweight and 12 obese NAFLD patients as well as 16 healthy controls. Sex and age did not differ between groups. Obese patients had a median BMI of 35.3 kg/m^2^, which corresponds to obesity grade II [28].

**Table 1:** Subject characteristics, basal and postprandial FGF19 serum concentrations

Variables	Control	Overweight	Obesity	*p*-value
N (men/women)	16 (7/9)	14 (8/6)	12 (6/6)	n.s.^a^
Age (years)	29.5 (24.0–53.0)	49.0 (38.8–57.3)	48.0 (37.0–57.3)	0.551^b^
BMI (kg/m^2^)	24.2 (21.8–26.6)	28.3 (26.3–29.2)	35.3 (32.7–39.0)	< 0.001^b^
FGF19 (pg/ml)				
t = 0 h	178.5 (101.0–257.0)^c^	127.5 (70.0–161.3)	116.0 (51.0–134.3)^c^	0.01^c^
t = 2 h	244.5 (161.5–377.5)^c^	163.0 (78.5–168.3)^c^	181.0 (85.3–393.0)	0.004^c^
t = 4 h	332.5 (202.0–590.8)	207.0 (112.5–365.0)	220.0 (138.8–385.3)	0.445^b^
t = 6 h	211.0 (165.3–296.3)	154.0 (124.0–254.0)	184.5 (110.5–274.3)	0.445^b^

All data are given as median (interquartile range)

^a^Chi-square-test

^b^Kruskal-Wallis-test

^c^Mann-Whitney-U-test

NAFLD was diagnosed by ultrasound and/or biopsy. In overweight and obese patients, the steatosis spectrum ranged from grade I, II and III to NASH and fibrosis. In the overweight group (*N* = 14), grade I liver steatosis was found in six patients (43%), grade II in five patients (36%), and grade III in one patient (7%). NASH was diagnosed in one and fibrosis stage II was documented in one patient. In the obese group (*N* = 12), two patients (17%) displayed liver steatosis grade I, four patients (33%) showed grade II, and three participants (25%) had grade III. In this group, NASH was found in one and fibrosis stage I also in one patient. In one obese study participant, liver status could not be assessed by ultrasound. Additional file 1: Table S1 lists the comorbidities in overweight and obese NAFLD patients. In overweight patients, hypercholesterolemia and in obese patients, arterial hypertension were the dominant concomitant diseases, respectively. Controls did not take any drugs regularly. Three overweight and eight obese patients were taking medications. These included antidiabetics, antihypertensives, thyroid hormones, analgesics, proton pump inhibitors, antidepressants, non-steroidal antirheumatics, corticoidsteroids and allopurinol, respectively.

#### Basal and postprandial FGF19 serum concentrations

**Fig. 1:**
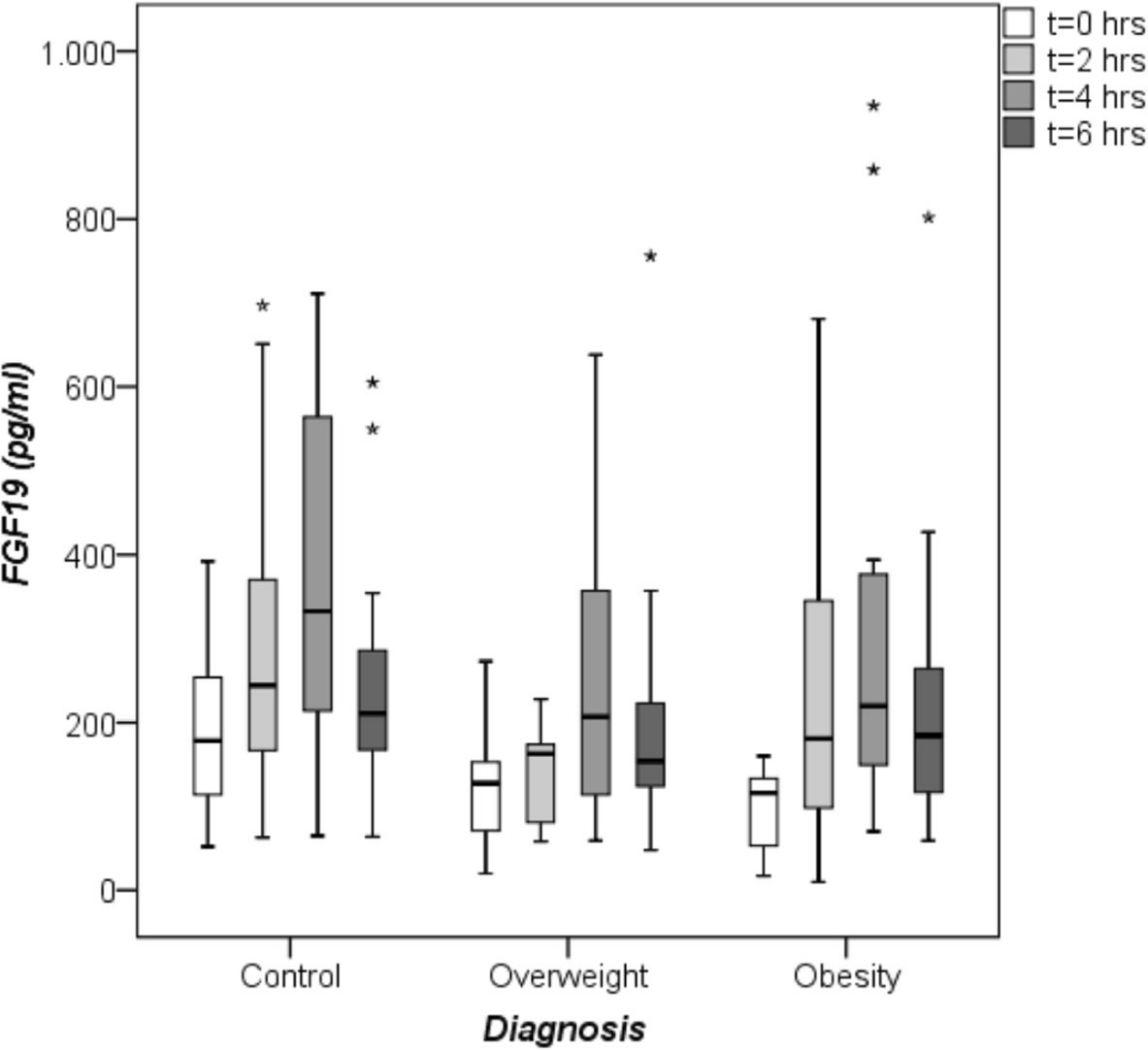
Fasting and postprandial FGF19 serum concentrations measured by quantitative sandwich enzyme-linked immunosorbent assay (ELISA). Comparison of FGF19 values between healthy controls (*N* = 16), overweight (*N* = 14) and obese (*N* = 12) patients with non-alcoholic fatty liver disease (NAFLD) at baseline (0 h), 2, 4 and 6 h after the oral fat tolerance test (OFTT). Significant difference between basal (0 h) FGF19 concentrations in controls and obese NAFLD patients [controls 178.5 (101.0–257.0) vs. obese 116.0 (51.0–134.3) pg/ml, medians (IQRs), *p* = < 0.05, Mann-Whitney-U-test). At 2 h, lower FGF19 values in overweight NAFLD patients in comparison to controls [overweight 163.0 (78.5–168.3) vs. controls 244.5 (161.5–377.5) pg/ml, medians (IQRs), *p* = 0.004, Mann-Whitney-U-test), * outlier

**Fig. 2:**
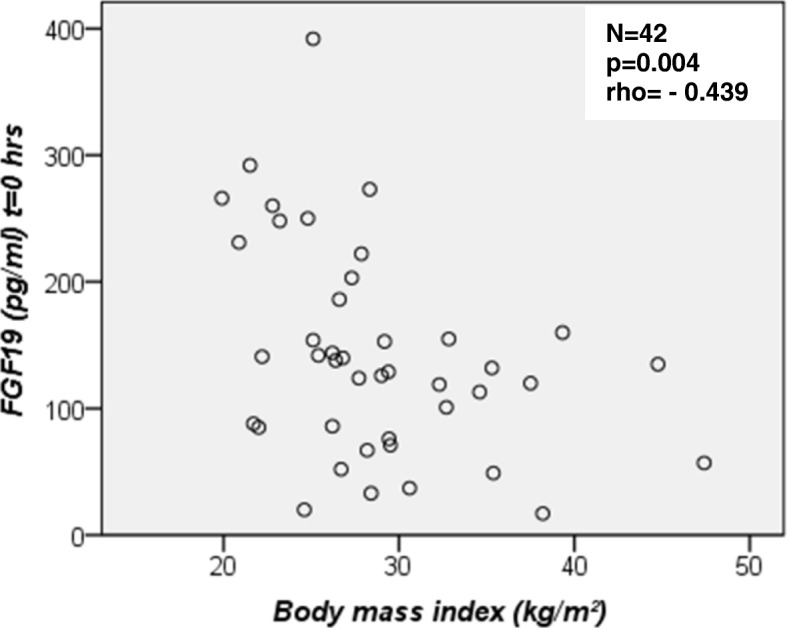
Fasting FGF19 serum concentrations versus body mass index (BMI) for all study subjects. FGF19 values correlated negatively with BMI. A scattered plot is shown and the Spearman’s correlation coefficient was calculated

In the total study group (*N* = 42), fasting FGF19 concentrations ranged from 17.0 to 392.0 pg/ml (median 133.5, IQR 82.8–190.3). Basal FGF19 values were significantly lower in obese NAFLD patients as compared to controls and tended to be lower in overweight NAFLD subjects, too (Table 1, Fig. 1). Basal FGF19 concentrations did not differ between sexes [women, median 126.0 (IQR 80.5–179.0) vs. men, median 138.0 (IQR 93.0–204.0) pg/ml]. Interestingly, fasting FGF19 concentrations were negatively correlated with BMI (Fig. 2).

After the OFTT, FGF19 concentrations increased in controls, overweight and obese patients (Table 1, Fig. 1). Of note, overweight patients displayed lowest FGF19 concentrations at all postprandial time points. Two hours after the OFTT, FGF19 levels ranged from 10.0 to 697.0 pg/ml (median 178.0, IQR 116.5–255.5). At this time, overweight NAFLD patients showed significantly lower FGF19 levels compared with controls (Fig. 1). The FGF19 maximum was found in all three groups after 4 h, with hormone levels ranging from 59.0 to 935.0 pg/ml (median 255.0, IQR 163.3–439.3). At 4 h, FGF19 was highest in controls and twice as high as at baseline. After 6 h, FGF19 values ranged from 48.0 to 802.0 pg/ml (median 189.0, IQR 136.0–269.0) and were still highest in controls.

Both women and men showed the FGF19 maximum at 4 h [women, median 270.0 (IQR 167.5–539.0) vs. men, median 231.0 (IQR 117.5–410.5) pg/ml] but postprandial levels were higher in women at all time points. Six hours after the oral fat challenge, FGF19 concentrations of both sexes tended to reach a significant difference [women, median 216.0 (IQR 145.0–390.5) vs. men, median 172.0 (IQR124.0–216.5) pg/ml, *p* = 0.051].

Mean FGF19_(0-6h)_-area and mean incremental area under the curve (AUC and IAUC) did not differ significantly between the groups (AUC controls: 1772.8 ± 766.6 vs. overweight: 1130.6 ± 590.0 vs. obese: 1469.3 ± 910.0 pg/ml/6 h; IAUC controls: 699.0 ± 383.7 vs. overweight: 573.3 ± 333.4 vs. obese: 921.5 ± 732.4 pg/ml). FGF19-AUC was highest in controls and lowest in overweight patients (*p* = 0.053); IAUC was higher in obese and lower in overweight patients in comparison to controls. FGF19-AUC and IAUC did not correlate with body weight-adjusted fat load. In addition, there was no association between FGF19-AUC and BMI; FGF19-IAUC tended to correlate with age (rho = 0.291, *p* = 0.062).

#### Basal and postprandial BA serum concentrations

**Fig. 3:**
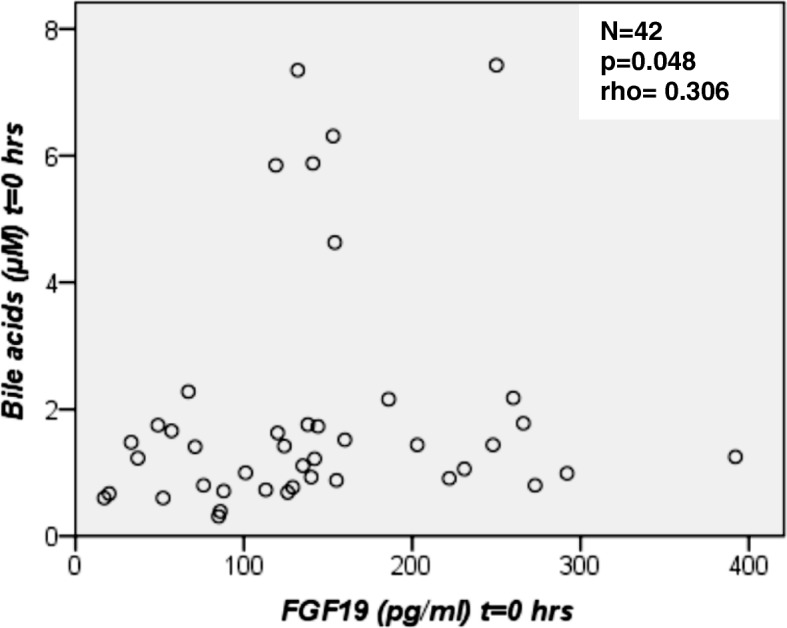
Fasting FGF19 serum concentrations versus fasting bile acid (BA) serum concentrations for all subjects (*N* = 42). There was a correlation between FGF19 and BA values. A scattered plot is shown and the Spearman’s correlation coefficient was calculated

Fasting and postprandial bile acid (BA) concentrations did not differ between overweight/obese NAFLD patients and controls. In all three groups, we observed a BA increase after the OFTT with a peak at 2 h (Table 2). Basal FGF19 concentrations correlated positively with basal BA values (Fig. 3).

**Table 2:** Basal and postprandial BA and C4 serum concentrations

Variables	Control	Overweight	Obesity	*p*-value
N (men/women)	16 (7/9)	14 (8/6)	12 (6/6)	n.s.^a^
Bile acids (μM)				
t = 0 h	1.1 (0.8–1.7)	1.5 (0.8–2.2)	1.4 (0.9–1.7)	0.343
t = 2 h	1.4 (1.1–4.7)	2.1 (1.2–3.8)	2.4 (1.6–4.1)	0.311
t = 4 h	1.1 (0.7–2.0)	2.0 (1.3–2.5)	2.0 (1.0–2.3)	0.155
t = 6 h	0.7 (0.5–1.6)	1.3 (0.8–2.0)	1.2 (0.8–1.7)	0.087
C4 (nM)				
t = 0 h	41.4 (7.0–69.2)	58.5 (12.8–91.6)	35.1 (1.2–72.3)	0.422
t = 2 h	28.1 (7.1–49.6)	43.1 (10.0–114.0)	35.7 (1.2–118.3)	0.765
t = 4 h	13.0 (6.8–44.7)	40.8 (21.1–99.3)	32.7 (3.3–108.0)	0.343
t = 6 h	11.8 (4.1–34.4)	40.7 (18.1–80.1)	28.6 (3.5–82.4)	0.445

All data are given as median (interquartile range), *p*-values: Kruskal-Wallis-test,^a^Chi-square-test

#### Basal and postprandial C4 serum concentrations

**Fig. 4:**
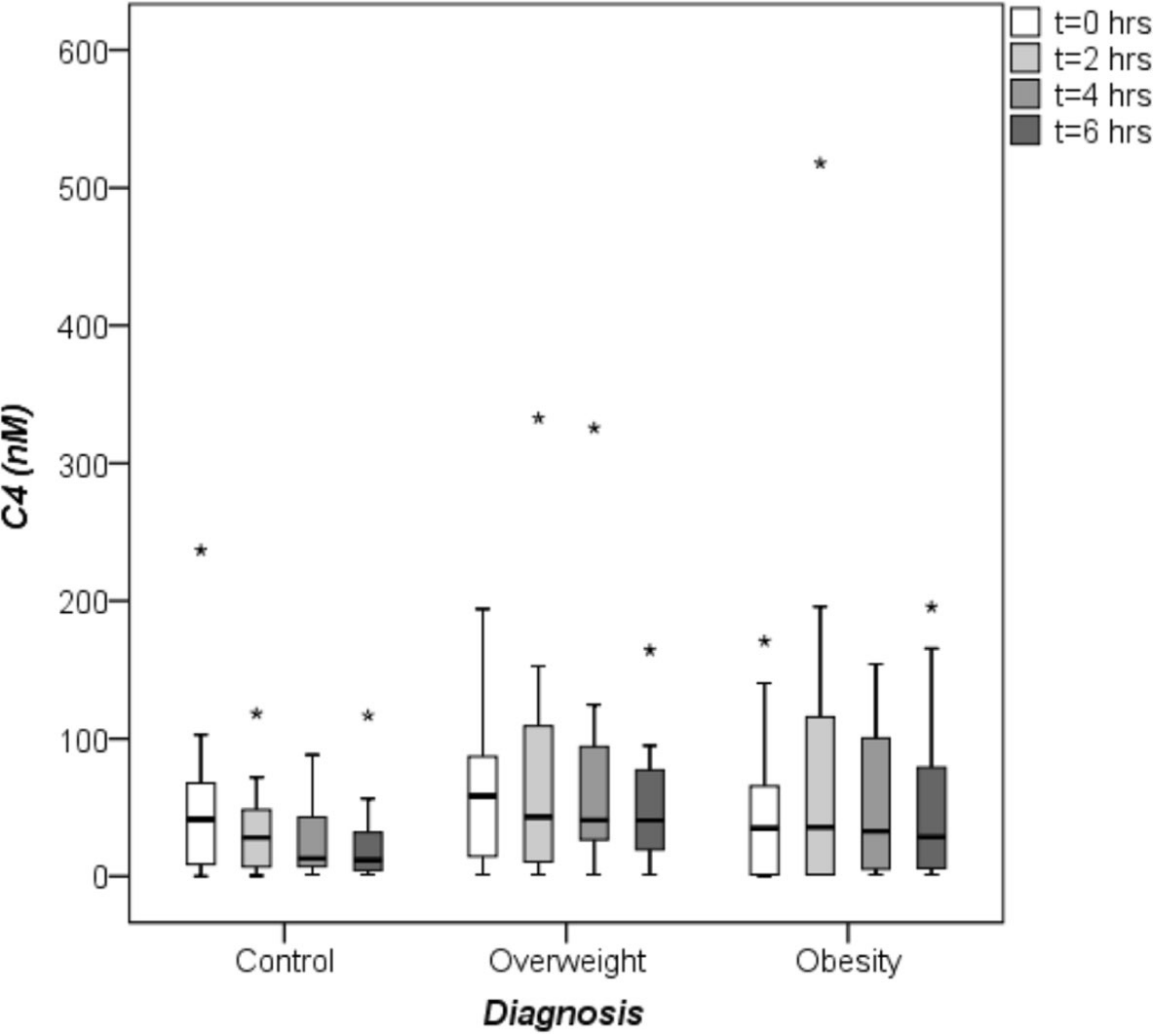
Fasting and postprandial C4 concentrations. C4, a valid marker of bile acid biosynthesis, was measured by high-performance liquid chromatography (HPLC). Comparison of C4 values between healthy controls (*N* = 16), overweight (*N* = 14) and obese (*N* = 12) patients with non-alcoholic fatty liver disease (NAFLD) at baseline (0 h), 2, 4 and 6 h after the oral fat tolerance test (OFTT). C4 concentrations did not differ between groups (Kruskal-Wallis-test),*outlier

**Fig. 5:**
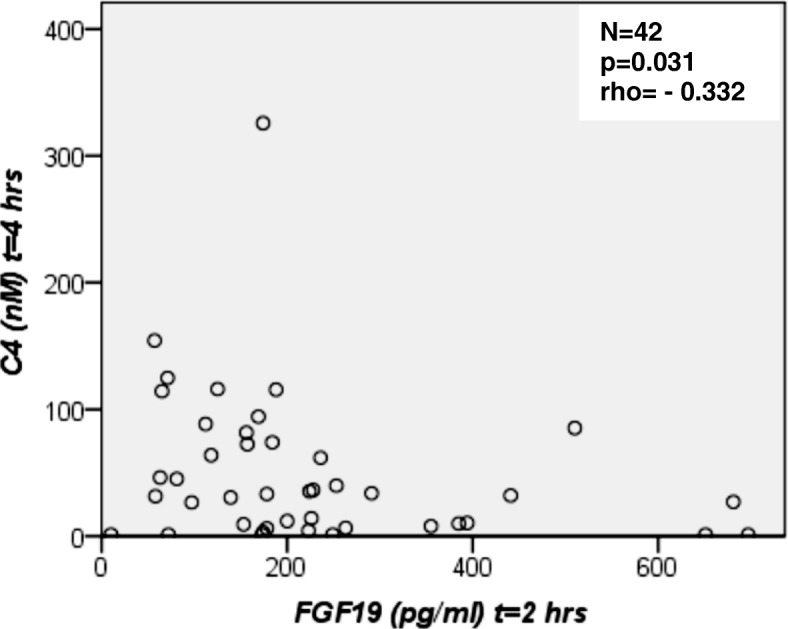
FGF19 serum concentrations at 2 h versus C4 values at 4 h after the oral fat tolerance test (OFTT) for all subjects (*N* = 42). A scattered plot is shown and the Spearman’s correlation coefficient was calculated

**Fig. 6:**
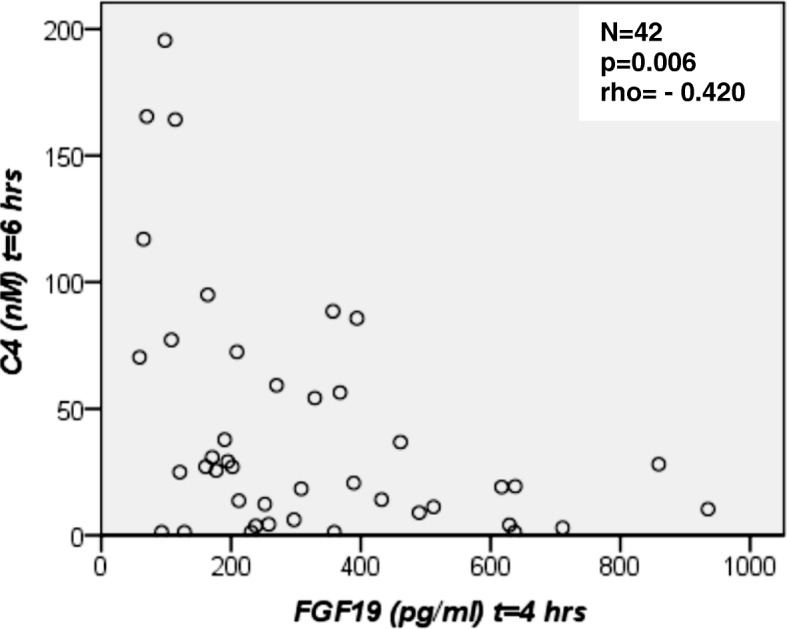
FGF19 serum concentrations at 4 h versus C4 values at 6 h after the oral fat tolerance test (OFTT) for all subjects (*N* = 42). A scattered plot is shown and the Spearman’s correlation coefficient was calculated

Fasting and postprandial C4 values did not differ significantly between study participants (Table 2, Fig. 4). At all postprandial time points, C4 concentrations were markedly lower in controls in comparison to overweight/obese NAFLD patients. C4 concentrations in overweight NAFLD patients remained unchanged for 4 h postprandially, despite increasing FGF19 values. In the total study group FGF19 concentrations at 2 h correlated negatively with C4 values at 4 h after the OFTT (Fig. 5). There was also an inverse correlation of FGF19 concentrations at 4 h and C4 values at 6 h after the OFTT in the study group (Fig. 6). These correlations were confirmed for the control group (Additional file 2: Figure S1, Additional file 3: Figure S2). In NAFLD patients, FGF19 concentrations did not correlate with C4 values.

## Discussion

The present study investigated serum FGF19, BA, and C4 profiles in overweight and obese NAFLD patients, in comparison to normal-weight healthy controls, after a body weight-adjusted oral fat load. The key findings of our study are (i) fasting FGF19 concentrations were significantly lower in obese (grade II) NAFLD patients as compared to controls, (ii) overweight NAFLD patients had significantly lower FGF19 concentrations 2 h after the fat load and lowest values at all postprandial time points and (iii) BAs increased during the OFTT but without changes in C4 levels.

Low FGF19 concentrations have been reported in metabolic syndrome [35], obesity [36] and type 2 diabetes [37, 38]. The findings by Jansen et al. [19] support our results, i.e. the presence of fatty liver disease in obesity is associated with lower fasting FGF19 concentrations. Also in obese children with NAFLD [20] and young obese NASH patients [39] lower fasting FGF19 values were detected whereas Schreuder et al. did not observe differences in fasting FGF19 concentrations between controls and obese NAFLD subjects [13]. In our study, BMI correlated negatively with fasting FGF19 serum concentrations. Our study was too small to correlate FGF19 levels with biopsy-proven severity of NAFLD. In this respect, inverse associations had been found in children [15, 40], but some studies did not find correlations between steatosis grade and FGF19 concentrations [13, 14].

To the best of our knowledge, we are the first group which established a body-weight adjusted oral fat tolerance test (OFTT) to stimulate BA secretion and subsequent FGF19 expression in overweight and obese NAFLD patients. Only a few studies report about postprandial FGF19 concentrations [13, 41, 42]. Similar oral fat load tests have been used for determining postprandial triglyceride concentrations, but to date no standard method is established [43]. Our controls showed the highest FGF19 values during OFTT, suggesting unimpaired intestinal FGF19 release. Remarkably, overweight NAFLD patients had significantly lower FGF19 concentrations 2 h after the fat load as compared to controls and lowest hormone values at all postprandial time points. In another study in healthy volunteers, oral fat load (75 g vegetable fat, mixture of Calogen®, sun flower and olive oil) also showed a stepwise increase of FGF19 between 2 and 4 h and a decrease at 6 h almost reaching fasting levels [41]. Schreuder et al. used whipped cream for their oral fat test in NAFLD patients [13]. The fat challenge was applied with 30 g cream (35% *w*/*v* fat) per m^2^ of body surface area. Single postprandial time points in plasma FGF19 concentrations did not differ between controls and NAFLD patients. Interestingly, the postprandial FGF19-IAUC was lower in NAFLD patients [13]. In our study, mean AUC and IAUC did not differ significantly, but AUC was highest in controls and lowest in overweight patients; in contrast, IAUC was highest in obese and lower in overweight patients in comparison to controls. In comparison to Schreuder’s study [13], our fat challenge was considerably higher, which could have contributed to differential findings at single postprandial time points and in FGF19-IAUC between controls and NAFLD patients.

Before our study started, we suspected that obese patients would have the highest energy and fat intake. Therefore we decided for a body weight-based fat load to adjust the OFTT to the patient’s eating behavior. To check our assumption, we used a 3-day nutritional protocol and calculated energy and macronutrients intake (data not shown). Since there was no significant difference between groups, we suppose under-reporting of food intake in our overweight and obese patients. Most researchers agree that the reported accuracy of food intake decreases with increasing BMI. A systematic review covering studies between 1982 and 2014 showed that a BMI > 30 kg/m^2^ is associated with significant under-reporting of food intake. These studies were mostly from Europe and North America [44]. For example, in morbid obese (BMI > 40 kg/m^2^) energy intake can reach more than 4000 kcal/day, with high fat intakes of about 40 to 57% of total energy intake [31].

In the present study, higher fat challenge in obese patients could explain their higher FGF19 values in comparison to overweight patients, which indicates that obese can compensate their low fasting FGF19 values by a high fat intake. Sonne and colleagues [42] found that FGF19 concentrations in patients with type 2 diabetes and healthy controls increased with increasing fat and decreasing carbohydrate content in liquid meals (500 kcal, 2.5 vs. 10.0 and 40.0 g fat). FGF19 values tended to be lower in type 2 diabetes patients compared with controls, but were not statistically significant.

Oral fat intake stimulates bile acid (BA) secretion. The entry of dietary fat in the duodenum causes gallbladder contraction and inflow of BA into the intestinal lumen. In the ileum, BA induce secretion of FGF19 that suppresses de novo BA synthesis in the liver [25]. In the present study fasting and postprandial BA concentrations did not differ between overweight/obese NAFLD patients and controls and C4 serum concentrations in NAFLD patients did not decrease as they did in controls. Therefore, the hepatic BA biosynthesis was presumably not repressed. One reason could be that CYP7A1 expression was insufficiently suppressed by FGF19 due to low fasting and postprandial FGF19 concentrations. Schreuder et al. [13] also reported an impaired hepatic response (no decline of C4) in NAFLD patients with insulin resistance. The pathomechanism(s) behind the observed blunted C4 response are unknown. One might speculate about impaired hepatic signaling after binding of FGF19 to the FGF receptor 4/βKlotho heterodimer or other factors within the fatty liver cell that might affect the feedback regulation of BA synthesis.

Our method of bile acid analysis did not yield complete profiles including conjugates with glycine or taurine. Thus, distinct differences in BA profiles between lean controls and NAFLD patients might have affected activation of FXR. This is supported by studies in Chinese children were levels of chenodeoxycholic acid (CDCA) were increased in the moderate/severe stage of NAFLD. The authors report, that decreased circulating level of deoxycholic acid (DCA) in children with mild NAFLD might have a negative effect on the activation of FXR, which subsequently triggers an increasing production of CDCA in patients with moderate to severe NAFLD [45]. In contrast, Jiao and colleagues [39] found increased DCA and decreased CDCA levels in NAFLD patients. In this context, changes in BA composition could also be one reason for altered intestinal FXR signaling, expressed as reduced FGF19 levels, in our overweight and obese NAFLD patients.

## Conclusions

Fasting FGF19 serum concentrations were lowest in obese NAFLD patients and highest in normal-weight healthy controls. Our body weight-adjusted oral fat challenge resulted in lowest FGF19 concentrations in overweight NAFLD patients at all postprandial time points. Overweight and obese NAFLD patients showed impaired FGF19 release in fasting and postprandial state. We assume that obese NAFLD patients were able to compensate their low fasting FGF19 values by a high (body weight-adjusted) oral fat intake. Reduced FGF19 values in overweight and obese NAFLD patients might reflect altered intestinal FXR signaling. How the hepatic receptor FGFR4 or its cofactor βKlotho modulate the hepatic response to FGF19 in NAFLD subjects should be examined further in functional studies.

## Additional files

Additional file 1: **Table S1.** Prevalence of comorbidities in overweight (*N* = 14) and obese (12) NAFLD patients. In overweight NAFLD subjects, hypercholesterolemia is the dominant concomitant disease. In obese NAFLD patients, arterial hypertension, hyperlipidemia and hyperuricemia are the most common comorbidities. (DOCX 18 kb)

Additional file 2: **Figure S1.** FGF19 serum concentrations at 2 h versus C4 values at 4 h after the oral fat tolerance test (OFTT) in controls (*N* = 16). (DOCX 36 kb)

Additional file 3: **Figure S2.** FGF19 serum concentrations at 4 h versus C4 values at 6 h after the oral fat tolerance test (OFTT) in controls (*N* = 16). (DOCX 37 kb)

## Abbreviations

BA: Bile acid
BMI: Body mass index
C4: 7α-hydroxy-4-cholesten-3-one
CDCA: Chenodeoxycholic acid
CYP7A1: Cholesterol 7α-hydroxylase
DCA: Deoxycholic acid
ELISA: Enzyme linked immunosorbent assay
FGF19: Fibroblast growth factor 19
FXR: Farnesoid X receptor
GC MS: Gas chromatography-mass spectrometry
HPLC: High-performance liquid chromatography
hrs: Hours
IQR: Interquartile range
NAFLD: Non-alcoholic fatty liver disease
NASH: Non-alcoholic steatohepatitis
OFTT: Oral fat tolerance test
