# Supplementary material for: Response of fibroblast growth factor 19 and bile acid synthesis after a body weight-adjusted oral fat tolerance test in overweight and obese NAFLD patients: a non-randomized controlled pilot trial
Source: BMC Gastroenterol. 2018 Jun 4;18:76. doi: 10.1186/s12876-018-0805-z (PMC5987457; doi:10.1186/s12876-018-0805-z)
Supplement: Supplementary file 1 — Table S1. Prevalence of comorbidities in overweight (N = 14) and obese (12) NAFLD patients. In overweight NAFLD subjects, hypercholesterolemia is the dominant concomitant disease. In obese NAFLD patients, arterial hypertension, hyperlipidemia and hyperuricemia are the most common comorbidities. (DOCX 18 kb) [file 12876_2018_805_MOESM1_ESM.docx]

**Table S1.** Prevalence of comorbidities in overweight and obese NAFLD patients.

| **Diagnosis** | **Overweight**  **(n=14)** | **Obesity**  **(n=12)** |
| --- | --- | --- |
| Arterial hypertension | - | 5 |
| Bronchial asthma | - | 2 |
| COPD | - | 1 |
| Diabetes mellitus type 2 | - | 1 |
| Gastroesophageal reflux disease | 1 | 2 |
| Hypercholesterolemia | 6 | 2 |
| Hyperlipidemia (mixed) | - | 3 |
| Hypertriglyceridemia | 1 | 1 |
| Hyperuricemia | - | 3 |
| Hypothyroidism | 1 | 1 |
| Impaired glucose tolerance | 2 | - |
| Sleep apnea syndrome | - | 2 |
| Turnersyndrome | 1 | - |
| None | 4 | 2 |
