# Supplementary material for: Response of fibroblast growth factor 19 and bile acid synthesis after a body weight-adjusted oral fat tolerance test in overweight and obese NAFLD patients: a non-randomized controlled pilot trial
Source: BMC Gastroenterol. 2018 Jun 4;18:76. doi: 10.1186/s12876-018-0805-z (PMC5987457; doi:10.1186/s12876-018-0805-z)
Supplement: Supplementary file 2 — Figure S1. FGF19 serum concentrations at 2 h versus C4 values at 4 h after the oral fat tolerance test (OFTT) in controls (N = 16). (DOCX 36 kb) [file 12876_2018_805_MOESM2_ESM.docx]

**N=16**

**p=0.004**

**rho= - 0.682**
